# Ferroptosis exacerbates the clonal deletion of virus-specific exhausted CD8^+^ T cells

**DOI:** 10.3389/fimmu.2024.1490845

**Published:** 2024-11-25

**Authors:** Qin Tian, Cheng Chen, Jinjin Lu, Xinyu Zheng, Xiuming Zhai, Yanping Yang, Ziyao Zhao, Jiangtao Hao, Ke Yang, Lilin Ye, Yifei Wang

**Affiliations:** ^1^ Dermatology Hospital, Southern Medical University, Guangzhou, China; ^2^ Guangdong Province Key Laboratory of Immune Regulation and Immunotherapy, School of Laboratory Medicine and Biotechnology, Southern Medical University, Guangzhou, Guangdong, China; ^3^ Institute of Immunology, Third Military Medical University, Chongqing, China; ^4^ Institute of Immunological Innovation and Translation, Chongqing Medical University, Chongqing, China; ^5^ School of Life Science, Chongqing University, Chongqing, China

**Keywords:** ferroptosis, exhausted CD8^+^ T cells, clonal deletion, T cell exhaustion, LCMV, chronic viral infection, GPX4, persistence

## Abstract

During chronic infection or tumorigenesis, persistent antigen stimulation contributes to the exhaustion of CD8^+^ T cells. Nevertheless, exhausted CD8^+^ T (T_EX_) cells still preserve certain effector function, and maintaining a reservoir of exhausted cells is of vital importance for virus elimination and tumor eradiation. Despite considerable work interrogating the rejuvenation of T_EX_ cells, mechanisms underpinning the clonal deletion of T_EX_ cells remain largely unexplored over the past decade. In this study, we employed mouse models of LCMV infection to demonstrate that excessive accumulation of lipid peroxidation rendered virus-specific T_EX_ cells to ferroptosis, which may correlate with enhanced mitochondria-derived oxidative stress and compromised activity of glutathione peroxidase 4 (GPX4). In addition, either incomplete or complete ablation of GPX4 resulted in exacerbated ferroptosis and aggravated shrunken population of virus-specific T_EX_ cells. On the other hand, inhibiting ferroptosis via administration of a ferroptosis inhibitor or overexpression of GPX4 greatly rectified the cell loss of virus-specific T_EX_ cells. Collectively, we disclosed ferroptosis as a crucial player in the clonal deletion of virus-specific T_EX_ cells and stressed the intervention of ferroptosis as a promising approach to optimize the longevity of virus-specific T_EX_ cells.

## 1 Introduction

During acute viral infection, naïve antigen-specific CD8^+^ T (T_N_) cells undergo clonal expansion and differentiate into effector CD8^+^ T (T_EFF_) cells, which further undergo apoptosis-driven contraction and transformed into a small population of long-lived memory CD8^+^ T (T_MEM_) cells (1, 2). Both T_EFF_ and T_MEM_ play a vital role in eliminating intracellular pathogens or malignant cells, providing robust and long-term protection to host (1, 2). However, in the context of chronic viral infection or tumor progression, T_EFF_ cells become exhausted due to the persistent antigen exposure and the presence of other repressive signals (3–5). Exhausted CD8^+^ T (T_EX_) cells manifest a hierarchical loss of effector functions, sustained expression of inhibitor receptors [e.g., programmed death-1 (PD-1), lymphocyte activation gene 3 (LAG-3), and CD39], and ultimately, a physical clonal deletion (3, 5). Given the recognition that apoptosis is the most well-known form of regulated cell death (RCD) to maintain the homeostasis of T cells and dominate the activation-induced cell death of T cells (6–8), apoptotic cell death is well acknowledged as the predominant type of RCD driving the clonal deletion of T_EX_ cells (9–11). With the growing knowledge of T-cell exhaustion mechanisms (3, 5, 12, 13) and newly emerging definitions of RCD (14–16), it seems plausible to speculate that the disrupted metabolism and unbalanced cell redox status in T_EX_ cells may potentially give rise to alternative forms of RCD beyond apoptosis.

Previously, we verified that mitochondrial reactive oxygen species (mtROS) generated by dysfunctional mitochondria and compromised antioxidative pathway were major inducements of ferroptosis in virus-specific memory CD4^+^ T cells (17). Ferroptosis was defined as an RCD triggered by excessive accumulation of lipid peroxidation in an iron-dependent manner (18–21). It was reported that the deletion of glutathione peroxidase 4 (GPX4), an intracellular glutathione peroxidase crucial for preventing lipid peroxidation (20, 22), led to compromised clonal expansion of CD8^+^ T cell during acute-resolved infections (23). Furthermore, in the settings of tumor progression, emerging evidence dictates that tumor-specific CD8^+^ T cells are susceptible to ferroptosis in exhaustion-promoting tumor microenvironment, ending up with compromised anti-tumor immunity (24–27). Although both chronic viral infection and tumorigenesis drive CD8^+^ T-cell exhaustion, the molecular signals and metabolic programming pathways in these two scenarios are believed to exhibit distinct features (12, 13, 28, 29). In the present study, we set out to investigate the potential role of ferroptosis in virus-specific T_EX_ cells in the context of chronic viral infection.

Taking advantage of the T-cell receptor (TCR) transgenic P14 mouse harboring TCR specific for lymphocytic choriomeningitis virus (LCMV) glycoprotein 33–41 (GP_33–41_) epitope, we evaluated the cellular lipid peroxidation in virus-specific CD8^+^ T cells during chronic viral infection. The observation that inhibiting ferroptosis substantially rectified the loss of T_EX_ cells further confirmed the contribution of ferroptosis to the clonal deletion of T_EX_ cells. Furthermore, our findings revealed that under the excessive mitochondria-derived oxidative stress, both the expression and enzymatic activity of GPX4 were compromised in T_EX_ cells, resulting in the occurrence of ferroptosis and ultimately the demise of virus-specific T_EX_ cells during chronic viral infection. Taken together, our data provide the first evidence of ferroptosis accounting for the clonal deletion of virus-specific T_EX_ cells during chronic viral infection. Additionally, we underscore the significance of ferroptosis inhibition in sustaining the virus-specific T_EX_ cell population, thereby offering a promising avenue for the treatment of chronic viral infection in clinical settings.

## 2 Materials and methods

### 2.1 Mice and viruses

*Gpx4* ^fl/fl^ (027964), ERT2Cre (010688), and wild-type (WT) CD45.2^+^ and CD45.1^+^ C57BL/6J (002014) congenic mice were obtained from the Jackson Laboratory. P14 transgenic mice with CD8^+^ T cells specific for LCMV GP_33–41_ epitope and LCMV strains Armstrong (Arm) and LCMV Clone-13 (Cl13) were provided by R. Ahmed (Emory University). Intraperitoneal (i.p.) injection of 2 × 10^5^ plaque-forming units (PFU) of the LCMV Arm established acute viral infection in mice. Intravenous (i.v.) injection of 2 × 10^6^ PFU of LCMV Cl13 established chronic viral infection in mice. Mice were infected at 6–10 weeks of age unless otherwise stated. All mice were housed in specific pathogen-free conditions at 25°C and 55% humidity on a 12-h dark/12-h light cycle.

### 2.2 Adoptive transfer and infection

For acute viral infection, a total of 2 × 10^4^ naive P14 (CD45.1^+^) cells were adoptively transferred into naive wild-type (WT) congenic recipient mice (CD45.2^+^). On the following day, the recipients were infected i.p. with 2 × 10^5^ PFU of the LCMV Arm. For chronic viral infection, a total of 2 × 10^3^ naive P14 (CD45.1^+^) cells were adoptively transferred into naive WT congenic recipient mice (CD45.2^+^). On the following day, the recipients were infected i.v. with 2 × 10^6^ PFU of LCMV Cl13. In some cases, *Gpx4* ^fl/fl^-ERT2Cre-P14 and WT-P14 cells were adoptively co-transferred at a ratio of 1:1 into congenic recipient mice and received LCMV Cl13 infection the next day.

### 2.3 Construction of mixed bone marrow chimeras

Bone marrow (BM) cells were collected from WT B6 mice (CD45.2^+^) and *Gpx4* ^fl/fl^-ERT2Cre (KO) mice (CD45.1^+^). A total of 6 × 10^6^ mixed BM cells were intravenously co-transferred at a KO to WT ratio of 3:7 into lethally irradiated WT recipients (CD45.2^+^), which received two doses of sublethal irradiation (550 rad). Chimeras were fed with antibiotic-containing water during the first 4 weeks and allowed a total of 8–10 weeks for reconstitution before infection.

### 2.4 Drug administration *in vivo* and *in vitro*

Tamoxifen (TAM, T5648, Sigma-Aldrich, Darmstadt, Germany) and its active metabolites, 4-hydroxy tamoxifen (4-OHT, H6278, Sigma-Aldrich, Darmstadt, Germany), were employed to inactivate GPX4 in T cells from *Gpx4* ^fl/fl^-ERT2Cre mice *in vivo* and *in vitro*, respectively. For *in vivo* GPX4 deletion, TAM was prepared and administrated as previously described (17). For *in vitro* GPX4 deletion, enriched CD8^+^ T cells from *Gpx4* ^fl/fl^-ERT2Cre-P14 mice were treated with 5 μM 4-OHT for 36 h before subjected to adoptive transfer.

Liproxstatin-1 (Lip-1; S7699, Selleck, Houston, TX, USA), a potent peroxyl radical scavenger to antagonize ferroptosis, was administrated i.p. at 30 mg/kg/day for 10 days. Mice in the control group received sham treatment with vehicle during the same period.

### 2.5 Flow cytometry

Flow cytometry data were acquired with a FACSCanto II or LSRFortessa (BD Biosciences, La Jolla, CA, USA) and were analyzed with Flowjo software (BD Biosciences, La Jolla, CA, USA). Major histocompatibility complex class I (H-2Db) tetramer specific for the LCMV epitope of GP_33–41_ was provided by a tetramer core facility of the US National Institutes of Health (Emory, Atlanta, GA, USA). The antibodies used in flow cytometry are listed as follows: anti-CD8 (clone 53-6.7), anti-CD45.1 (clone A20), anti-CD45.2 (clone 104), anti-CD44 (clone IM7), anti-TCR Vα2 (clone B20.1), anti-PD-1 (clone 29F.1A12), anti-LAG3 (clone C9B7W), and anti-Ki-67 (clone 11F6) were purchased from Biolegend (San Diego, CA, USA); anti-TIM3 (clone RMT3-23) and anti-CD39 (clone 24DMS1) were purchased from eBioscience (San Diego, CA, USA); anti-GPX4 (clone EPNCIR144]) was purchased from Abcam (Cambridge, UK). Cell surface staining was performed in FACS buffer. Staining of GPX4 was performed with a BD Cytofix/Cytoperm kit (554714, BD Biosciences, La Jolla, CA, USA) after cell surface staining. Staining of Ki-67 was performed with a Foxp3/Transcription kit (554714, BD Biosciences, La Jolla, CA, USA) after cell surface staining. Staining of apoptosis was conducted using a commercial apoptosis detection kit (559763, BD Pharmingen, San Diego, CA, USA) following manufacturer’s instructions.

### 2.6 Molecular probes or mitochondrial dye staining

Cells were stained with cell surface markers before being subjected to molecular probe or mitochondrial dye staining in R10 (RPMI medium containing 10% fetal bovine serum) at 37°C under 5% CO_2_ for 30 min, unless otherwise stated. To detect mitochondrial mass, cells were further stained with 100 nM MitoTracker Green (M7514, Invitrogen, Carlsbad, CA, USA) in PBS (calcium and magnesium free). To measure lipid peroxidation, cells were further stained with 5 μM BODIPY 581/591 C11 (BODIPY-C11, D3861, Invitrogen, Carlsbad, CA, USA) or 4 μM Liperfluo (L248, Dojindo, Mashiki, Tabaru, Japan) in PBS. In some scenario, cells were treated with PBS or hydrogen peroxide (H_2_O_2_, 1 mM) for 1 h at room temperature before staining with BODIPY-C11. For ROS or mtROS detection, cells were loaded with 5 μM CellROX Deep Red (C10422, Invitrogen, Carlsbad, CA, USA) or 5 μM MitoSOX (M36008, Invitrogen, Carlsbad, CA, USA). To determine mitochondria membrane potential changes, cells were stained with 100 nM TMRE (87917, Sigma-Aldrich, Darmstadt, Germany). Cells were then resuspended in FACS buffer and subjected to flow cytometric analysis.

### 2.7 Fluorescence-activated cell sorting

Fluorescence-activated cell sorting (FACS) was performed on a BD FACSAria III cell sorter (BD Biosciences, La Jolla, CA, USA) at the Clinical Medical Research Center of Southwest Hospital, Third Military Medical University. T_N_ (CD44^lo^CD62L^hi^, or CD44^lo^CD62L^hi^Vα2^+^), T_EFF_ and T_MEM_ (CD44^hi^, or CD44^hi^CD45.1^+^), and T_EX_ (CD44^hi^PD-1^hi^, or CD44^hi^PD-1^hi^CD45.1^+^) cells were sorted at indicated timepoints post LCMV Arm or Cl13 infection after CD8^+^ T cell enrichment via depletion of cells positive for lineage markers through the use of biotin-conjugated antibodies [anti-CD4 (RM4-5, Biolegend, San Diego, CA, USA), anti-B220 (RA3-6B2, Biolegend, San Diego, CA, USA), anti-CD25 (PC61, Biolegend, San Diego, CA, USA), anti-CD11b (M1/70, Biolegend, San Diego, CA, USA), anti-CD11c (N418, Biolegend, San Diego, CA, USA), anti-Gr-1 (RB6-8C5, Biolegend, San Diego, CA, USA), anti-TER119 (TER-119, Biolegend, San Diego, CA, USA), anti-CD49b (DX5, Invitrogen, Carlsbad, CA, USA), and anti-NK1.1 (PK136, Biolegend, San Diego, CA, USA)] coupled to BeaverBeads Mag500 Streptavidin Matrix (22302, Beaver, Suzhou, Jiangsu, China). The population purity after cell sorting was verified >95% in all experiments. Sorted CD8^+^ T cells were then subjected to RNA analysis, immunoblot analysis, and glutathione peroxidase activity assay.

### 2.8 Glutathione peroxidase activity assay

FACS-sorted T_N_, T_EFF_, T_MEM_, and T_EX_ cells were used to conduct glutathione peroxidase activity assay using a Glutathione Peroxidase Assay kit (ab102530, Abcam, Cambridge, UK) according to manufacturer’s instructions.

### 2.9 Real-time quantitively PCR

Total RNA was sorted from FACS-sorted T_N_, T_EFF_, T_MEM_ and T_EX_ cells using a Trizol-based approach, followed by reverse transcription (RT) via a commercial RT kit (R212, Vazyme, Nanjing, Jiangsu, China). The resulting cDNA was analyzed with a SYBR Green PCR kit (208054, Qiagen, Tegelen, Hulsterweg, Netherlands) on a CFX96 Touch Real-Time system (Bio-Rad, Hercules, CA, USA). Here are the primers used in the study: *Gpx4* (*Gpx4*-F, 5′-CGCGATGATTGGCGCT-3′; *Gpx4*-R, 5′-CACACGAAACCCCTGTACTTATCC-3′) and house-keeping gene *Hprt* (*Hprt*-F, 5′-TCAGTCAACGGGGGACATAAA-3′; *Hprt*-R, 5′-GGGGCTGTACTGCTTAACCAG-3′).

### 2.10 Viral load determination

Viral load in specific tissues (spleen and brain) of LCMV Cl13-infected mice were determined as previously described with minor modifications (30). Briefly, indicated tissues were weighted and homogenized with Trizol for RNA extraction, followed by RT using the aforementioned commercial RT kit (R212, Vazyme) with an LCMV-specific glycoprotein (GP) primer (GP-R, 5′-GCAACTGCTGTGTTCCCGAAAC-3′). The qPCR assay was conducted with LCMV GP-specific primer pair (GP-F, 5′-CATTCACCTGGACTTTGTCAGACTC-3′; GP-R, 5′-GCAACTGCTGTGTTCCCGAAAC-3′). The viral load was determined and presented as PFU per gram as previously described (31).

### 2.11 Gene set enrichment analysis

We retrieved publicly accessible RNA-sequencing data of T_EFF_ or T_EX_ P14 cells from the mice infected with LCMV Arm or Cl13 (32), from GEO database (GEO accessible number, GSE119942). The data were subjected to gene set enrichment analysis (GSEA) (Broad Institute) with gene sets from the Molecular Signatures Database or published data (17, 22, 33, 34) using the official GESA application or R script.

### 2.12 Western blot

FACS-sorted T_EFF_, T_MEM_, and T_EX_ cells were subjected to Western blot (WB) as previously described with minor modifications (17). Briefly, sorted cells were lysed in RIPA buffer, and thereafter, extracted protein samples were boiled with SDS sample buffer. Equal amounts of protein were separated by 15% SDS-PAGE and transferred onto a PVDF membrane (ISEQ00010, Millipore, Burlington, MA, USA). After blocking with 5% bovine serum albumin, membranes were probed overnight with primary antibodies anti-GPX4 (ab125066, Abcam, Cambridge, UK) or anti-β-Actin (4970S, Cell Signaling Technology, Danvers, MA, US) at 4°C. The membranes were probed with horseradish peroxidase-conjugated antibody to rabbit IgG (7074, Jackson ImmunoResearch Laboratories, West Grove, PA, USA) after cleanup of unbounded primary antibody. Membranes were then washed four times before visualized with a SuperSignal West Femto Maximum Sensitivity Substrate (34094, Thermo Fisher Scientific, Waltham, MA, USA) using a Fusion imaging system (FUSION Solo S, VILBER, Marne-la-vallée, Ile-de-France, France).

### 2.13 Retroviral constructs and transduction

Previously, retroviruses that overexpress any isoform of *Gpx4* have been constructed based on their intracellular location, including MIGRA-cGPX4 (cGPX4-OE), MIGRA-mGPX4 (mGPX4-OE), and MIGRA-nGPX4 (nGPX4-OE) (17). Briefly, cGPX4 locates in the cytoplasm and nucleus, mGPX4 locates in the mitochondria, and nGPX4 locates in the nucleus (35). Retroviruses knocking down *Gpx4* were constructed based on the backbone pMKO.1-GFP (shCTRL). Each construct incorporated the sequences of a unique shRNA targeting Gpx4 (pMKO.1-shGPX4-GFP, shGPX4 for short). Sequences of shRNA are as follows:

shGPX4-1: 5′-GACGTAAACTACACTCAGCTA-3′;

shGPX4-2: 5′-CAAATGGAACTTTACCAAGTT-3′;

shGPX4-3: 5′-CCCACTGTGGAAATGGATGAA-3′;

shGPX4-4: 5′-GCCAGGAAGTAATCAAGAAAT-3′.

All sequences were verified by DNA sequencing (Tsingke Biotech, Beijing, China). Retroviruses were packaged with 293T cells using package plasmid pCL-Eco and indicated retroviral constructs. *In vivo* activated P14 cells by GP_33–41_ peptide were spin transduced for 120 min at 37°C by centrifugation (2,100 rpm) with freshly collected retroviral supernatants, 8 μg/ml polybrene (H9268, Sigma-Aldrich, Darmstadt, Germany), and 20 ng/ml IL-2 (130-098-221, Miltenyi Biotec, Bergisch Gladbach, Germany). Then, the transduced P14 cells were sorted and transferred into recipient mice, followed by infection of the hosts with LCMV Cl13 or Arm the next day.

### 2.14 Data analysis and statistics

Statistical analysis was conducted with Prism 9.2.0 software (GraphPad). Flow cytometric data analysis was conducted using FlowJo software (BD Biosciences, La Jolla, CA, USA). Quantifications of WB results were conducted using Fiji image analysis software (ImageJ 1.54g, NIH). For comparisons between two independent groups, significance was determined by two-tailed unpaired and paired Student’s *t*-tests as indicated in different settings. A *p <* 0.05 was considered statistically significant. Asterisks were used to indicate significance: *p* < 0.05*, *p* < 0.01**, *p* < 0.001***, and *p* < 0.0001****, and ns, not significant.

## 3 Results

### 3.1 Lipid peroxidation is accumulated in virus-specific CD8^+^ T cells during chronic viral infection

**Figure 1:**
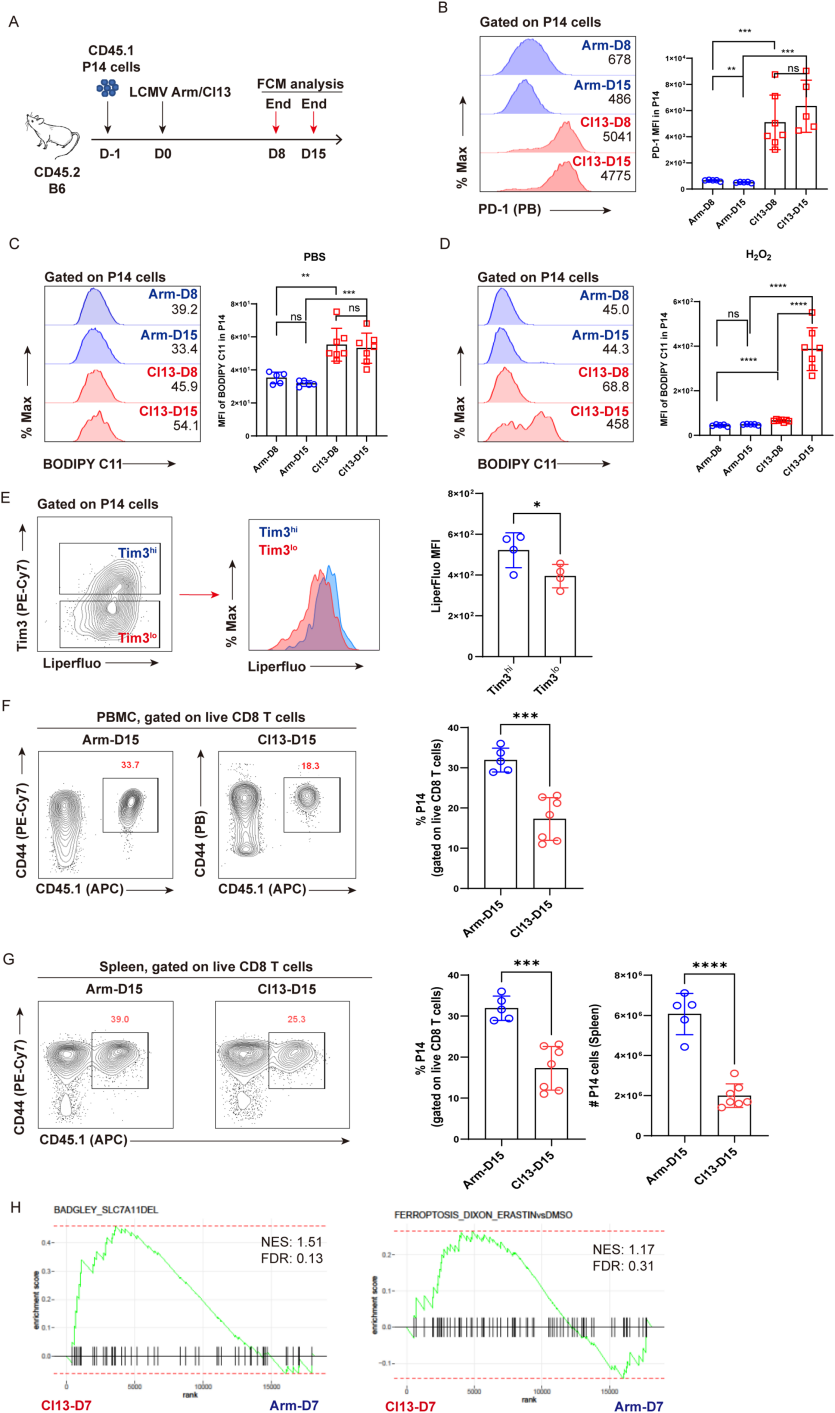
Lipid peroxidation is accumulated in virus-specific T_EX_ cells. **(A)** Experimental setup. CD45.1^+^ naive P14 cells were transferred into CD45.2^+^ congenic recipient mice, followed by LCMV Arm or Cl13 infection. Recipients were euthanized at D8 and D15 and subjected to flow cytometric analysis. **(B)** Representative histogram plots and statistics of PD-1 expression on P14 cells. Representative histogram plots and statistics of BODIPY-C11 MFI on P14 cells under treatment with PBS **(C)** or H_2_O_2_ **(D)**. **(E)** Representative contour plot and histogram plot, and statistics of Liperfluo MFI on Tim3^hi^ or Tim3^lo^ P14 cells. **(F)** Representative contour plots and statistics of the frequency of P14 cells in PBMCs at D15 post Arm or Cl13 infection. **(G)** Representative contour plots and statistics of the frequency and number of P14 cells in spleens at D15 post Arm or Cl13 infection. **(H)** GSEA plots for gene signatures associated with ferroptosis. **(B–G)** Data were collected from four to seven mice per group with two independent experiments. Statistical differences were calculated by unpaired t-test. *p < 0.05, **p < 0.01, ***p < 0.001, and ****p < 0.0001; ns, not significant.

We first leveraged two LCMV strains, Cl13 and Arm, and P14 mice expressing TCR specific recognizing the LCMV GP_33–41_ epitope to facilitate the investigation of virus-specific CD8^+^ T cells. CD45.1^+^ naive P14 cells isolated from P14 mice were adoptively transferred into congenic CD45.2^+^ recipient C57BL/6 (B6) mice, followed by infection with LCMV Cl13 or LCMV Arm 1 day later to establish either chronic or acute viral infection, respectively (**Figure 1A**). This experimental setup enables. At indicated timepoints post-infection (D8 and D15), parallel comparison of virus-specific T_EFF_ and T_EX_ cells was conducted via flow cytometric analysis. The gating strategy of P14 cells is illustrated as in **Supplementary Figure S1**. Exhausted state of T_EX_ P14 cells during chronic infection was verified by significantly higher expression of PD-1 compared to that in T_EFF_ P14 cells (**Figure 1B**). As expected, T_EX_ P14 cells from chronically infected mice manifested increased apoptosis (Annexin V^+^ or Annexin V^+^7-AAD^−^, **Supplementary Figures S2A, B**) and enhanced cell death (7-AAD^+^, **Supplementary Figure S2C**) relative to T_EFF_ P14 cells in acutely resolved infected mice.

Furthermore, we applied BODIPY-C11, a molecular probe specifically detecting the accumulation of peroxided lipids, to examine the ferroptosis of virus-specific CD8^+^ T cells. Compared to functional T_EFF_ P14 cells, T_EX_ P14 cells displayed a pronounced accumulation of peroxided lipids, irrespective of whether they were under physiological conditions (PBS-treated) (**Figure 1C**) or under excessive oxidative stress (H_2_O_2_ treated) (**Figure 1D**), indicating the occurrence of ferroptosis in exhausted P14 cells at 8 and 15 days post-infection (8 dpi and 15 dpi). Furthermore, a progressively accumulated lipid peroxidation was observed in T_EX_ cells at 15 dpi in comparison to that at 8 dpi under excessive oxidative stress (**Figure 1D**), implying that terminally differentiated T_EX_ P14 cells may be more susceptible to ferroptosis. In addition, the intensity of Liperfluo in terminally exhausted (Tim3^hi^) population was found to be notably higher than in the less exhausted (Tim3^lo^) counterpart (**Figure 1E**). In this scenario, we observed that mice from the Cl13–D15 group displayed a significantly lower frequency and number of P14 cells in both PBMCs (**Figure 1F**) and spleens (**Figure 1G**), compared to those from the Arm-D15 group.

Moreover, using publicly available bulk RNA sequencing data (32), we performed GSEA to compare the transcriptional differences between virus-specific T_EFF_ and T_EX_ cells with respect to ferroptosis-associated signatures (17, 22, 33, 34). GSEA results revealed that gene signatures of ferroptosis were more enriched in T_EX_ cells relative to T_EFF_ cells. (**Figure 1H**).

To conclude, our findings indicate that in addition to apoptosis, ferroptosis may also be responsible for the clonal depletion of virus-specific T_EX_ cells.

### 3.2 Ferroptosis inhibition alleviates the reduction of T_EX_ P14 cells

**Figure 2:**
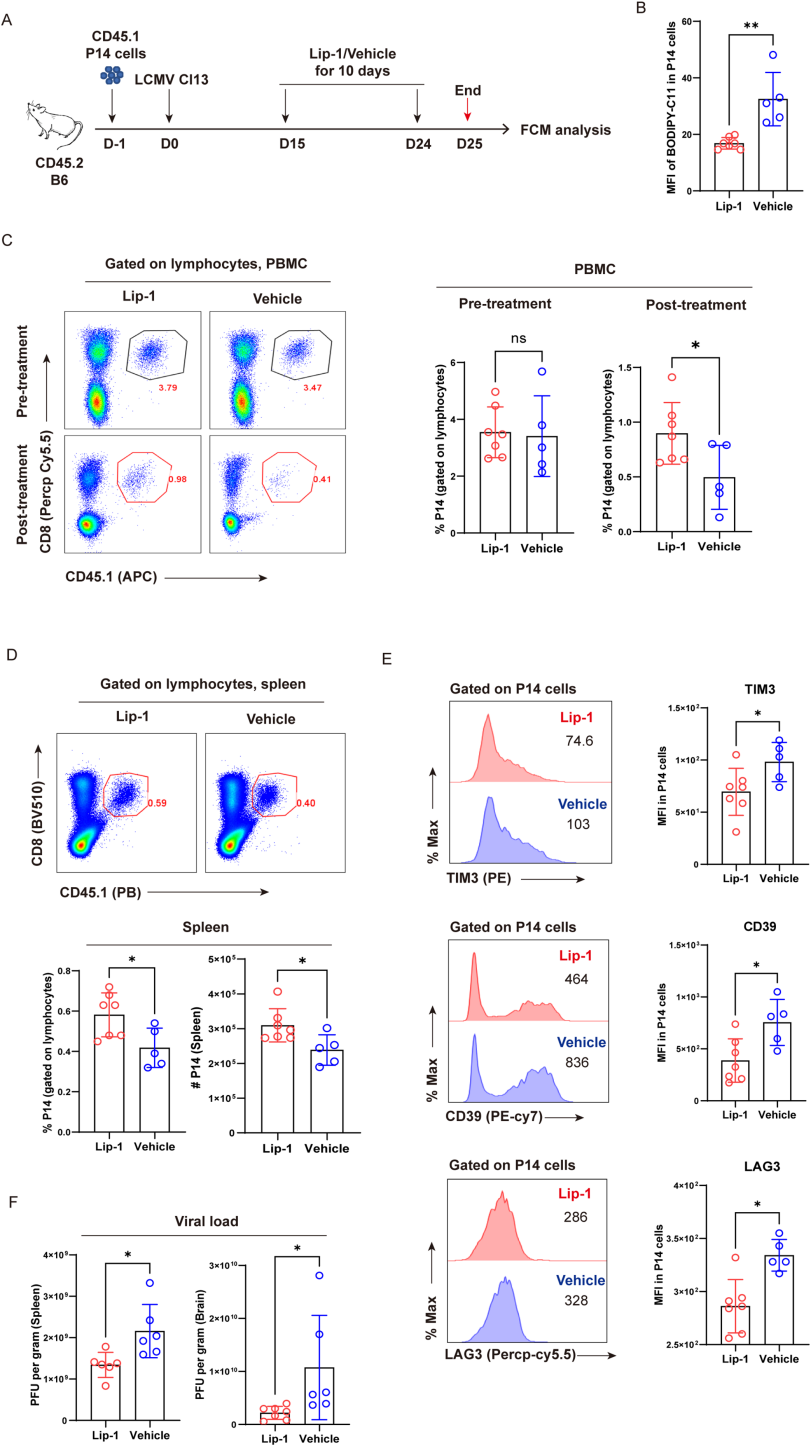
Selective inhibition of ferroptosis alleviates the exhaustion of virus-specific T_EX_ cells. **(A)** Experimental setup. CD45.1^+^ naive P14 cells were transferred into CD45.2^+^ congenic recipient mice, followed by LCMV Cl13 infection. Recipients received Lip-1 or vehicle treatment from 15 dpi to 24 dpi and were euthanized at 25 dpi, then subjected to flow cytometric analysis. **(B)** Statistics of MFI of BODIPY-C11 in P14 cells. **(C)** Representative pseudo-color plots and statistics of the frequency of P14 cells in PBMCs, pre- and post-treatment. **(D)** Representative pseudo-color plots and statistics of the frequency and number of P14 cells in spleens. **(E)** Representative histogram plots and statistics of MFI of inhibitory receptors in P14 cells. **(F)** RT-qPCR results showing viral load of lymphoid (spleen) and non-lymphoid (brain) tissues from recipient mice upon Lip-1 or vehicle treatment. **(B–F)** Data were collected from five to seven mice per group with two independent experiments. Statistical differences were calculated by unpaired t-test. *p < 0.05 and **p < 0.01; ns, not significant.

To further confirm that ferroptosis accounted for the reduction in T_EX_ cells, we set out to testify whether pharmacological inhibition of ferroptosis could alleviate the decline of virus-specific T_EX_ cells. CD45.1^+^ naive P14 cells were adoptively transferred into congenic CD45.2^+^-recipient B6 mice, and the hosts were subsequently infected with LCMV Cl13 the following day to establish chronic viral infection. Recipient mice were administrated with either the ferroptosis inhibitor liproxstatin-1 (Lip-1) or vehicle via the i.p. route between 15 dpi and 24 dpi and were subsequently euthanized at 25 dpi (**Figure 2A**). We first verified that Lip-1 treatment could efficiently inhibit ferroptosis by reducing the accumulation of lipid peroxides (**Figure 2B**). Compared to vehicle treatment, Lip-1 treatment resulted in markedly elevated frequency of P14 cells in PBMCs (~ 2-fold, 25 dpi), which was comparable in both Lip-1 and vehicle groups prior to treatment (15 dpi) (**Figure 2C**). In comparison to vehicle treatment, a significantly higher proportion and larger absolute number of P14 cells were consistently observed in the spleens of mice from the Lip-1 treatment group (~1.5-fold, 25 dpi) (**Figure 2D**). Moreover, Lip-1 treatment significantly restrained the terminal differentiation of T_EX_ P14 cells by reducing the expression of inhibitory receptors, including TIM3, CD39, and LAG3 (**Figure 2E**). Accordingly, the administration of Lip-1 resulted in a notable reduction in the viral load in both lymphoid (spleen) and non-lymphoid (brain) tissues (**Figure 2F**).

Collectively, we corroborate the role of ferroptosis in driving the clonal deletion of T_EX_ cells and unravel that selective inhibition of ferroptosis considerably increases both the frequency and cell number of virus-specific T_EX_ cells in the settings of chronic viral infection and ultimately results in a better viral control of hosts.

### 3.3 Ferroptosis is driven by mitochondrial dysfunction and oxidative stress in T_EX_ P14 cells

**Figure 3:**
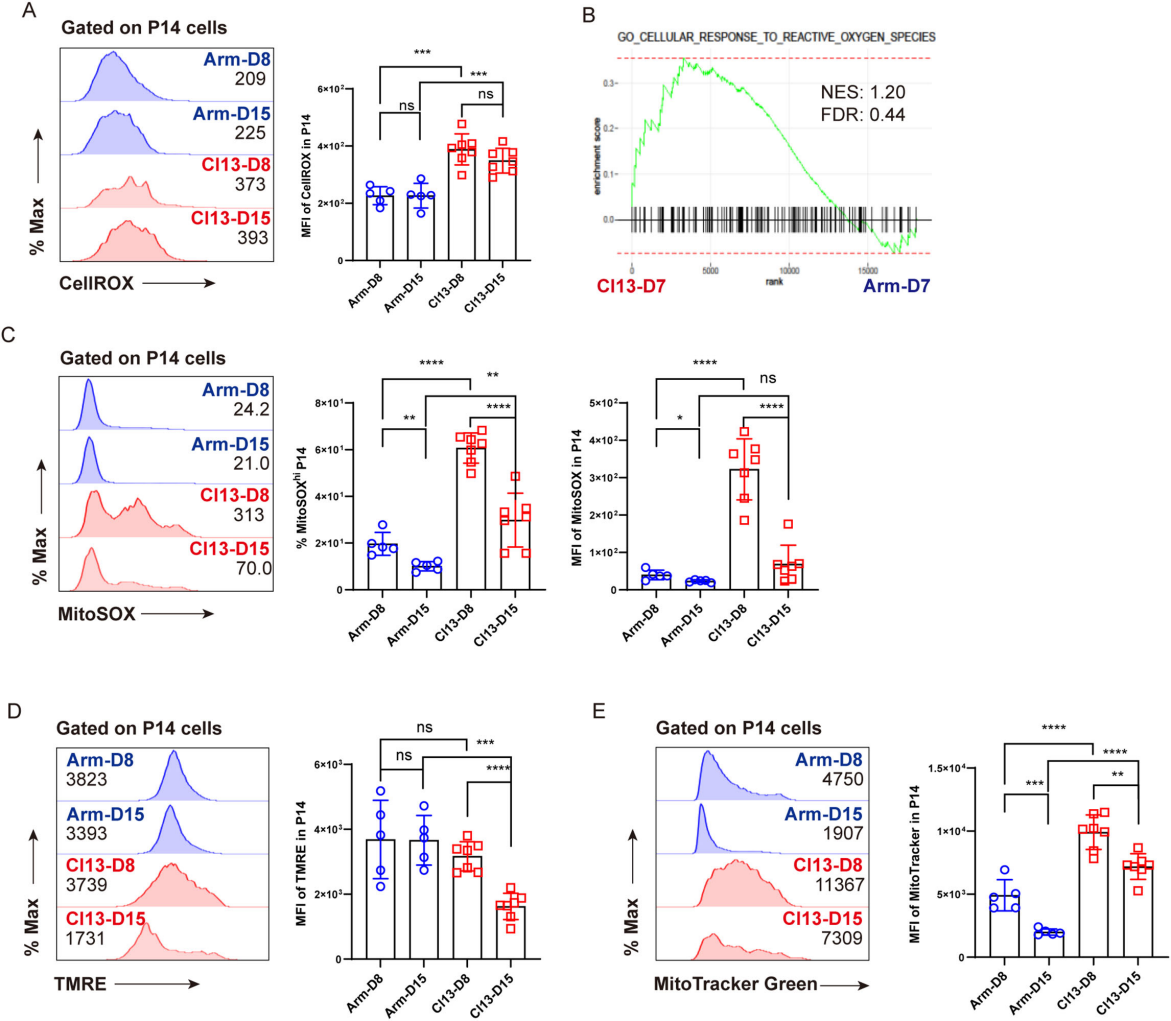
Imbalanced redox states and dysfunctional mitochondria in virus-specific T_EX_ cells. Experimental setup is illustrated as in **Figure 1A**. **(A)** Representative histogram plots and statistics of CellROX MFI on P14 cells. **(B)** GSEA plots for gene signatures associated with cellular response to ROS. **(C)** Representative histogram plots and statistics of MitoSOX MFI, and frequency of MitoSOX^hi^ P14 on P14 cells. **(D)** Representative histogram plots and statistics of TMRE MFI on P14 cells. **(E)** Representative histogram plots and statistics of MitoTracker Green MFI on P14 cells. **(A, C–E)** Data were collected from five to seven mice per group with two independent experiments. Statistical differences were calculated by unpaired t-test. *p < 0.05, **p < 0.01, ***p < 0.001, and ****p < 0.0001; ns, not significant.

Compared to functional T_EFF_ cells, T_EX_ cells generally manifested imbalanced redox states and dysfunctional mitochondria (4, 36). We speculated that aberrant redox balance and dysfunctional mitochondria may trigger ferroptosis in T_EX_ cells. To this end, we implemented the acute and chronic LCMV infection model as in **Figure 1A** and set out to analyze the differences in cellular oxidative stress and functional markers of mitochondria between T_EFF_ and T_EX_ cells using flow cytometry. In comparison with T_EFF_ P14 cells, T_EX_ P14 cells manifested a markedly elevated intensity of the fluorescent probe CellROX, indicative of a heightened level of intracellular ROS (**Figure 3A**). GSEA results also revealed enrichment of signatures associated with the cellular response to ROS in T_EX_ P14 cells (**Figure 3B**). Furthermore, as indicated by the fluorescent probe MitoSOX, an increased mtROS was observed in T_EX_ P14 cells rather than in T_EFF_ P14 cells (**Figure 3C**). Aberrant accumulation of ROS, especially mtROS, suggested a poor cellular fitness and mitochondrial dysfunction in T_EX_ P14 cells (36). Furthermore, we monitored the mitochondrial membrane potential (MMP) of T_EX_ P14 cells using the fluorescent probe tetramethylrhodamine ethyl ester (TMRE) and discovered a remarkably decreased MMP of T_EX_ P14 cells at later timepoints (Cl13 D15), echoing the increased apoptosis and the mitochondrial dysfunction in T_EX_ P14 cells (**Figure 3D**). In addition, we measured mitochondrial mass using the fluorescent probe MitoTracker Green, finding a marked accumulation of dysfunctional mitochondria in T_EX_ P14 cells (**Figure 3E**).

Taken together, we speculated that aberrant redox balance and accumulated dysfunctional mitochondria may contribute, at least in part, to the ferroptosis of virus-specific T_EX_ cells during LCMV Cl13 infection.

### 3.4 The expression and activity of GPX4 are both impaired in T_EX_ cells

**Figure 4:**
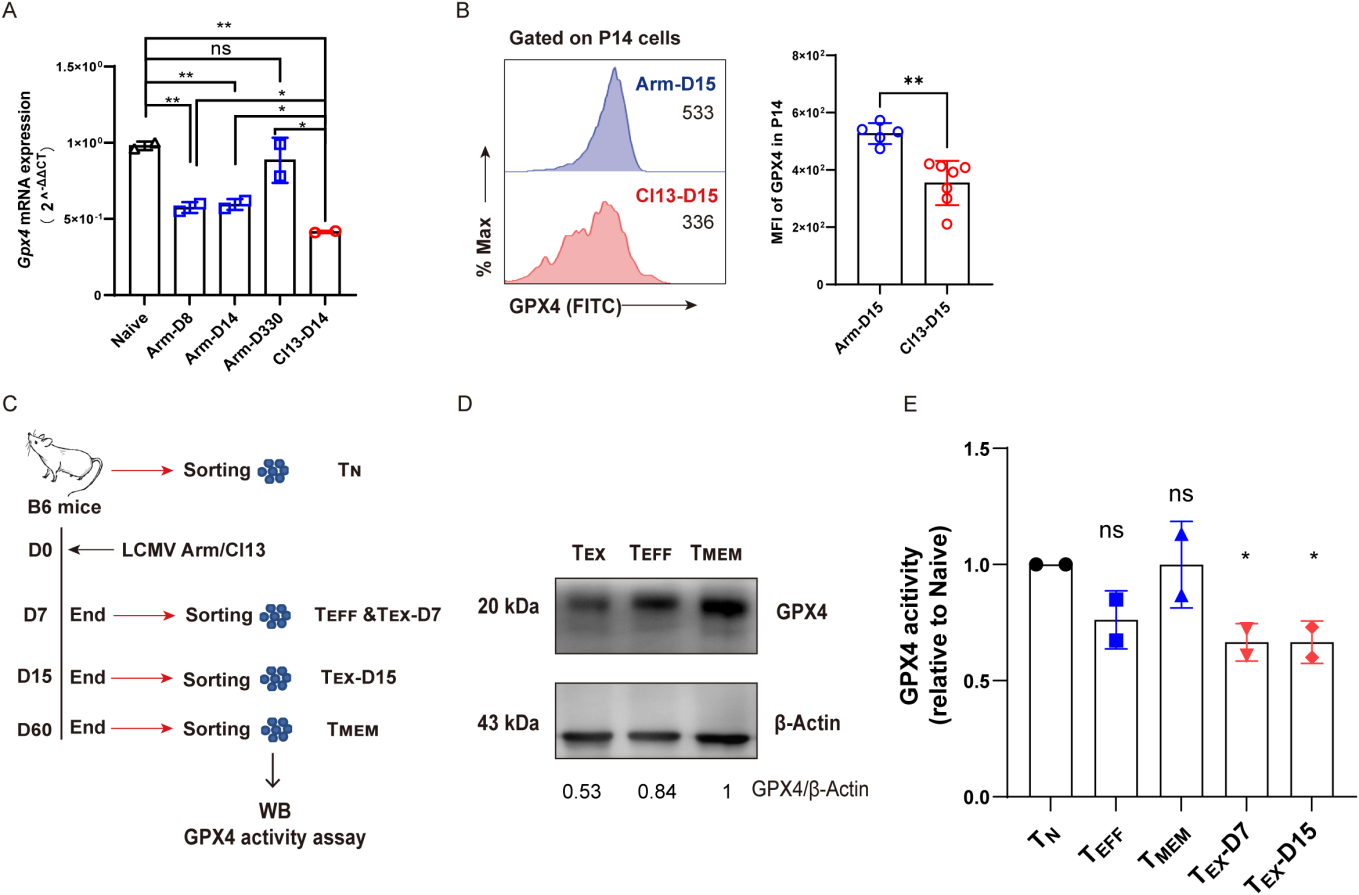
Compromised transcription, translation, and enzymatic activity of *Gpx4* in T_EX_ cells. **(A)** Naive (T_N_), effector (T_EFF_, Arm-D8, and Arm-D14), memory (T_MEM_, Arm-D330), and exhausted (T_EX_, Cl13-D14) P14 cells were sorted and subjected to RT-qPCR detecting *Gpx4* mRNA levels. **(B)** Flow cytometric analysis of GPX4 expression between T_EFF_ (Arm-D15) and T_EX_ (Cl13-D15) P14 cells. **(C)** Experimental setup. WT B6 mice were infected at various timepoints and euthanized at the same day to sort T_N_ (D0, CD44^lo^CD62L^hi^), T_EFF_ (Arm-D7, CD44^hi^), T_MEM_ (Arm-D60, CD44^hi^), and T_EX_ (Cl13-D7 and Cl13-D15, CD44^hi^ PD1^hi^) cells to conduct WB and GPX4 activity assay. **(D)** WB analysis of GPX4 expression among T_EFF_, T_MEM_, and T_EX_ (Cl13-D15) cells, and the number below the gel denotes the ratio of GPX4 to β-Actin. **(E)** Relative GPX4 activity of T_EFF_, T_MEM_, and T_EX_ (Cl13-D7 and Cl13-D15) cells. **(A, E)** The presented data are representative of two independent experiments (*n* = 2 replicates). **(B)** Data were collected from five to seven mice per group with two independent experiments. **(A, B, E)** Statistical differences were calculated by unpaired t-test. *p < 0.05 and **p < 0.01; ns, not significant.

As a critical enzyme preventing the accumulation of lipid peroxidation, GPX4 plays a pivotal role in antagonizing ferroptosis (22, 23). We set out to evaluate the content and activity of GPX4 in T_EX_ cells in comparison to other T-cell subsets during viral infection. To this end, T_N_, T_EFF_ (Arm-D8 or Arm-D15), T_MEM_ (Arm-D330), and T_EX_ (Cl13-D14) P14 cells were sorted to conduct real-time quantitively PCR (RT-qPCR) assay to detect the transcription levels of the *Gpx4* gene. We observed that *Gpx4* transcription was significantly repressed in T_EX_ P14 cells compared to those in T_N_, T_EFF_, and T_MEM_ P14 cells (**Figure 4A**). In addition, we examined the protein level of GPX4 using both flow cytometry and Western blot and discovered that the protein expression of GPX4 was severely decreased in T_EX_ cells compared to that in T_EFF_ or T_MEM_ cells (**Figures 4B–D**). Moreover, a significant reduction in GPX4 enzymatic activity was observed in T_EX_ cells (Cl13-D7 or Cl13-D15) in comparison to that in T_N_ cells (**Figure 4E**). In contrast, no significant difference was observed between T_EFF_ and T_N_ cells or between T_MEM_ and T_N_ cells (**Figure 4E**).

To conclude, the expression of *Gpx4* and the enzymatic activity of GPX4 were markedly impeded in T_EX_ cells during chronic viral infection, which may contribute to the ferroptotic cell death of T_EX_ cells.

### 3.5 Knockdown of GPX4 results in decreased T_EX_ P14 cells

**Figure 5:**
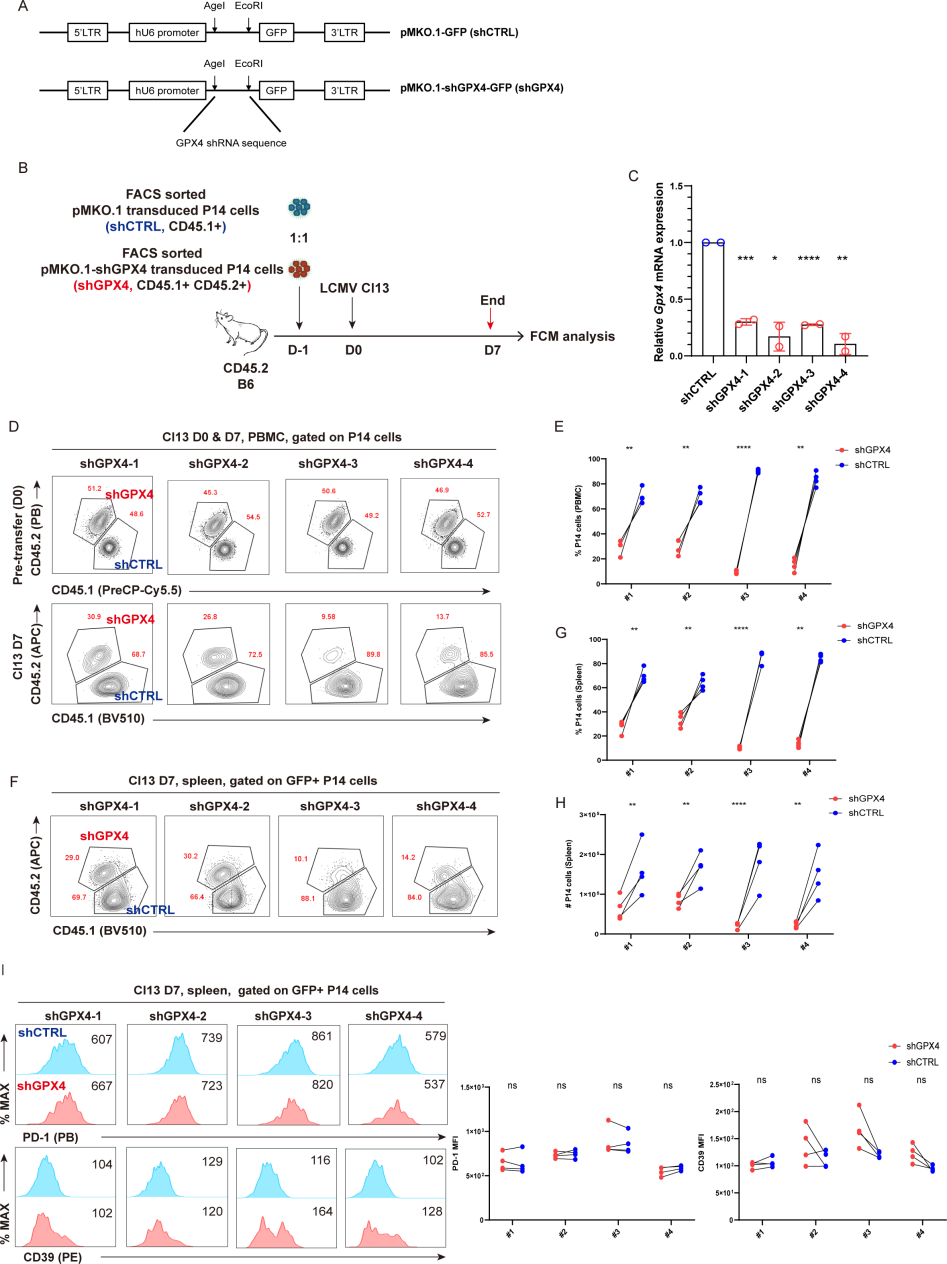
GPX4-KD renders the reduction of T_EX_ P14 cells. **(A)** Illustration of retroviral constructs. pMKO.1-shGPX4-GFP was constructed by inserting *Gpx4* shRNA sequences into the MCS of backbone pMKO.1-GFP using restriction enzyme *Age*I and *Eco*RI. **(B)** FACS sorted shGPX4- and shCTRL-transduced P14 cells were adoptively co-transferred into recipient mice, followed by LCMV Cl13 infection 1 day later. Mice were euthanized at 7 dpi, and both PBMCs and splenocytes were subjected to flow cytometric analysis. **(C)** RT-qPCR verifying the knockdown efficiency of shGPX4 in P14 cells. Representative contour plots **(D)** and statistics **(E)** of frequency of P14 cells in PBMCs, in the settings of GPX4-KD or not. Representative contour plots **(F)** and statistics of frequency **(G)** and cell number **(H)** of P14 cells in spleen, in the settings of GPX4-KD or not. **(I)** Representative histogram plots and statistics of MFI of inhibitory receptors in splenic P14 cells, in the settings of GPX4-KD or not. **(C)** The presented data are representative of two independent experiments (*n* = 2 replicates). **(E, G–I)** Data were collected from four mice per group with two independent experiments. Statistical differences were calculated by unpaired t-test **(C)** and paired t-test **(E, G–I)**. *p < 0.05, **p < 0.01, ***p < 0.001, and ****p < 0.0001; ns, not significant.

To assess the significance of GPX4 in the prolongation of T_EX_ cells during chronic viral infection, we utilized a retrovirus-mediated gene knockdown (KD) approach to specifically knock down GPX4 in P14 cells. Four retroviral constructs were created based on the backbone pMKO.1-GFP (shCTRL). Each construct carries a unique shRNA targeting *Gpx4* (pMKO.1-shGPX4-GFP, shGPX4 for short). This methodology is illustrated in detail in **Figure 5A**. Both shCTRL-transduced P14 cells (CD45.1^+^) and shGPX4-transduced P14 cells (CD45.1^+^CD45.2^+^) were sorted and adoptively co-transferred at a ratio of 1:1 into the recipient mice (CD45.2^+^), which were subsequently infected i.v. with LCMV Cl13 1 day later. Both PBMCs and splenocytes from recipients were subjected to flow cytometric analysis at 7 dpi (**Figure 5B**). We first validated that all the four shRNAs can efficiently reduce *Gpx4* transcription with a percent decrease ranging from 70% to 90% (**Figure 5C**). We also validated that the abovementioned four shRNAs can efficiently reduce *Gpx4* protein levels from the *in vitro* results (**Supplementary Figures S3A, B**). We then confirmed that GPX4-KD resulted in a substantial reduction in P14 cell frequency in PBMCs (**Figures 5D, E**). Additionally, both the frequency and cell number of P14 cells were observed to decline consistently in spleens from recipients in GPX4-KD group at 7 dpi (**Figures 5F–H**). However, GPX4-KD did not alter the expression of inhibitory receptors, such as PD-1 and CD39 (**Figure 5I**).

We also checked whether knockdown of *Gpx4* impaired cell proliferation *in vitro*. The ratio of shGPX4-transduced P14 cells to shCTRL-transduced P14 cells was significantly decreased at D4 compared to that at D0 (**Supplementary Figure S3C**). Moreover, GPX4-KD resulted in a decreased frequency of Ki-67^+^ P14 cells and a reduced expression of Ki-67 (**Supplementary Figure S3D**). To summarize, GPX4-KD significantly impaired cell proliferation of P14 cells *in vitro.*

In conclusion, we affirmed that the partial loss of GPX4 expression via GPX4-KD in P14 cells significantly exacerbated the decline of virus-specific T_EX_ cells.

### 3.6 Acute deletion of GPX4 aggravates the decay of T_EX_ P14 cells

**Figure 6:**
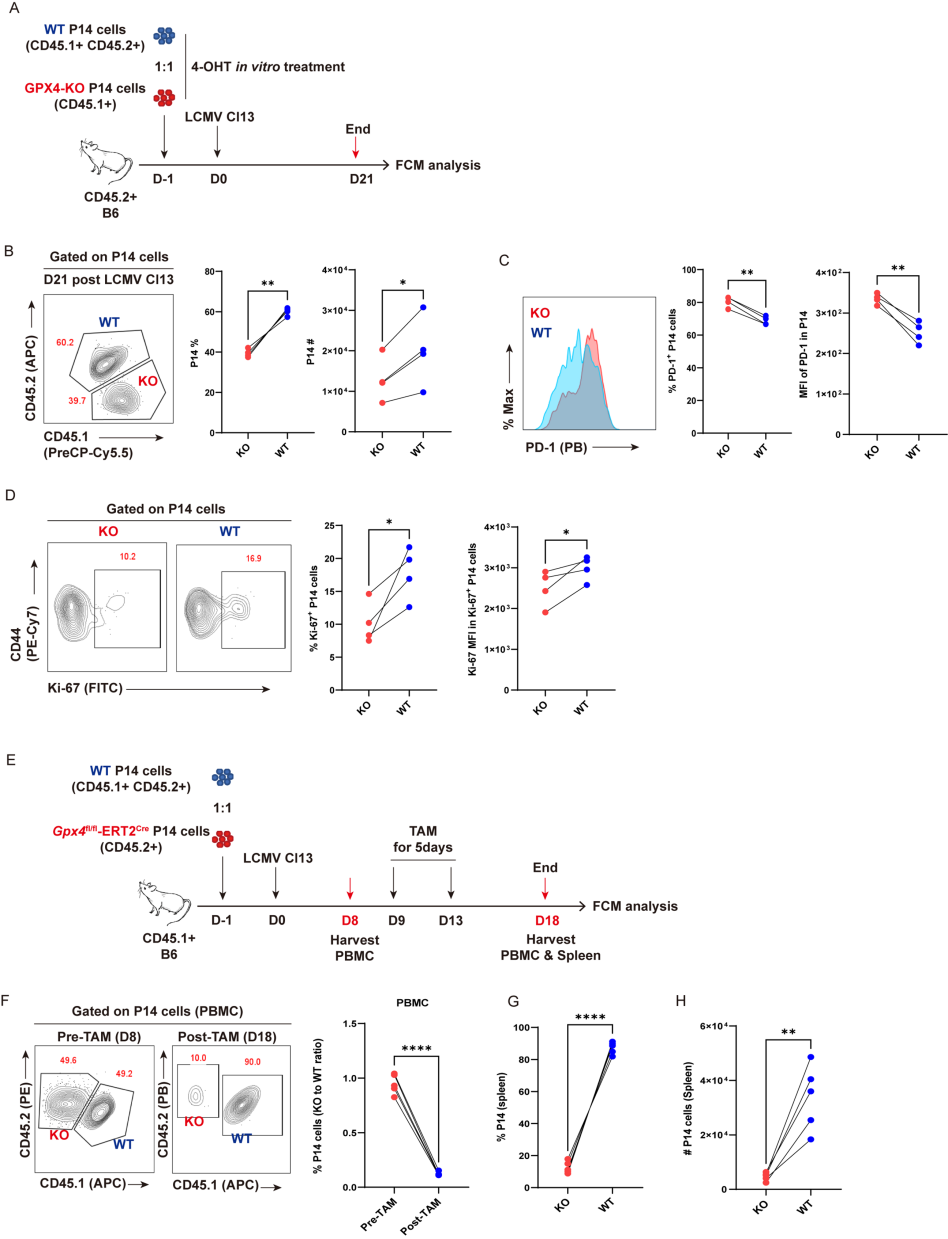
GPX4-KO diminishes T_EX_ P14 cells. **(A–D)** GPX4-KO before LCMV Cl13 infection. **(A)** Experimental setup. 4-OH-treated Naïve CD45.1^+^CD45.2^+^ WT P14 cells (WT) and CD45.1^+^ *Gpx4* ^fl/fl-^ERT2Cre-P14 cells (KO) were adoptively co-transferred into CD45.2^+^ congenic recipient mice, followed by LCMV Cl13 infection. Recipients will be euthanized at D21, and splenocytes were subjected to flow cytometric analysis. **(B)** Representative contour plot and statistics of frequency and cell number of splenic P14 cells at 21 dpi. **(C)** Representative histogram plot and frequency of PD-1^+^ P14 cells and PD-1 MFI in P14 cells. **(D)** Representative contour plots and frequency of Ki-67^+^ P14 cells and Ki-67 MFI in Ki-67^+^ P14 cells. **(E–H)** GPX4-KO post establishment of T-cell exhaustion. **(E)** Experimental setup. Naive CD45.1^+^CD45.2^+^ WT P14 cells (WT) and CD45.1^+^ *Gpx4* ^fl/fl-^ERT2Cre-P14 cells (KO) were adoptively co-transferred into CD45.2^+^ congenic recipient mice, followed by LCMV Cl13 infection. Recipients were administrated with tamoxifen from 9 dpi to 13 dpi, and euthanized at 18 dpi, then both PBMCs and splenocytes were subjected to flow cytometric analysis. **(F)** Representative contour plot and statistics of frequency of P14 cells in PBMCs, pre- and post-tamoxifen. Statistics of frequency **(G)** and cell number **(H)** of KO and WT P14 cells at 18 dpi in spleens. **(B–D, F–H)** Data were collected from four to five mice per group with three independent experiments. Statistical differences were calculated by paired t-test. *p < 0.05, **p < 0.01, and ****p < 0.0001.

To further substantiate the indispensability of anti-ferroptosis function of GPX4 in the persistence of virus-specific T_EX_ cells, *Gpx4* ^fl/fl-^ERT2Cre-P14 (KO-P14) mouse was bred to facilitate the tamoxifen (TAM)/4-OHT-inducible knockout of *Gpx4* in virus-specific P14 cells. P14 cells from KO-P14 mice were treated with 5 μM 4-OHT *in vitro* for 36 h to acutely delete *Gpx4*. Meanwhile, P14 cells from WT-P14 mice were subjected to 4-OHT treatment as a control (**Figure 6A**). We adoptively co-transferred 4-OHT-treated WT-P14 cells and 4-OHT-treated KO-P14 cells at a ratio of 1:1 into the recipient mice, then infected these mice with LCMV Cl13. Mice were euthanized at 21 dpi and subjected to flow cytometric analysis (**Figure 6A**). Phenocopied with T_EX_ P14 cells by GPX4-KD, GPX4-KO markedly reduced both the frequency and cell number of T_EX_ P14 cells (**Figure 6B**). We testified that GPX4-KO significantly increased BODIPY-C11 intensity (**Supplementary Figures S4A, B**), indicative of an augmented ferroptosis. Consequently, a pronounced ferroptosis brought a more exhausted state of P14 cells, as evidenced by a heightened frequency of PD-1^+^ P14 cells and upregulated PD-1 expression (**Figure 6C**). In addition, we observed a significant reduction in cell proliferation in KO-P14 cells relative to WT cells, indicated by a diminished frequency of Ki-67^+^ P14 cells and deceased Ki-67 expression (**Figure 6D**), underscoring a vital role of GPX4 in the renewal and persistence of T_EX_ P14 cells.

Next, we sought to analyze the impact of deletion of GPX4 by means of TAM administration in established T_EX_ P14 cells during chronic viral infection (**Figure 6E**). We validated that GPX4-KO faithfully increased the intensity of BODIPY-C11 in T_EX_ P14 cells at 5 days post TAM treatment (**Supplementary Figure S4C**). As expected, the frequency of KO-P14 cells was markedly diminished in PBMCs relative to that in WT cells, resulting in a notable reduction in the KO to WT ratio (**Figure 6F**). Consistently, both the frequency and cell number of KO-P14 cells (KO) exhibited a pronounced decrease in spleens compared to those in WT counterparts (**Figures 6G, H**).

Moreover, to validate the role of GPX4 and ferroptosis in endogenous T_EX_ cells, we constructed bone marrow chimeras (BMCs) by transferring bone marrow cell mixtures from *Gpx4* ^fl/fl^-ERT2Cre (CD45.1^+^) and B6 (CD45.2^+^) mice into the lethally irradiated recipient mice (CD45.2^+^). Eight weeks post-reconstitution, BMCs were infected with LCMV Cl13, followed by administration of TAM from 8 dpi to 12 dpi, and mice were euthanized at 8 days post TAM treatment for flow cytometric analysis (**Supplementary Figure S5A**). We observed that the amount of BODIPY-C11 was more enriched in KO polyclonal PD1^hi^ CD44^hi^ T_EX_ cells than in WT ones (**Supplementary Figure S5B**), coinciding with a dramatically decreased ratio of KO to WT in PBMC-derived polyclonal PD1^hi^ CD44^hi^ CD8^+^ T cells and in endogenous virus-specific splenic CD44^hi^ GP33^+^ CD8^+^ T cells (**Supplementary Figures S5C, D**).

To summarize, we depicted that GPX4 was intrinsically indispensable for the persistence of T_EX_ cells by antagonizing ferroptosis.

### 3.7 Enforced overexpression of GPX4 mitigates the decay of T_EX_ P14 cells

**Figure 7:**
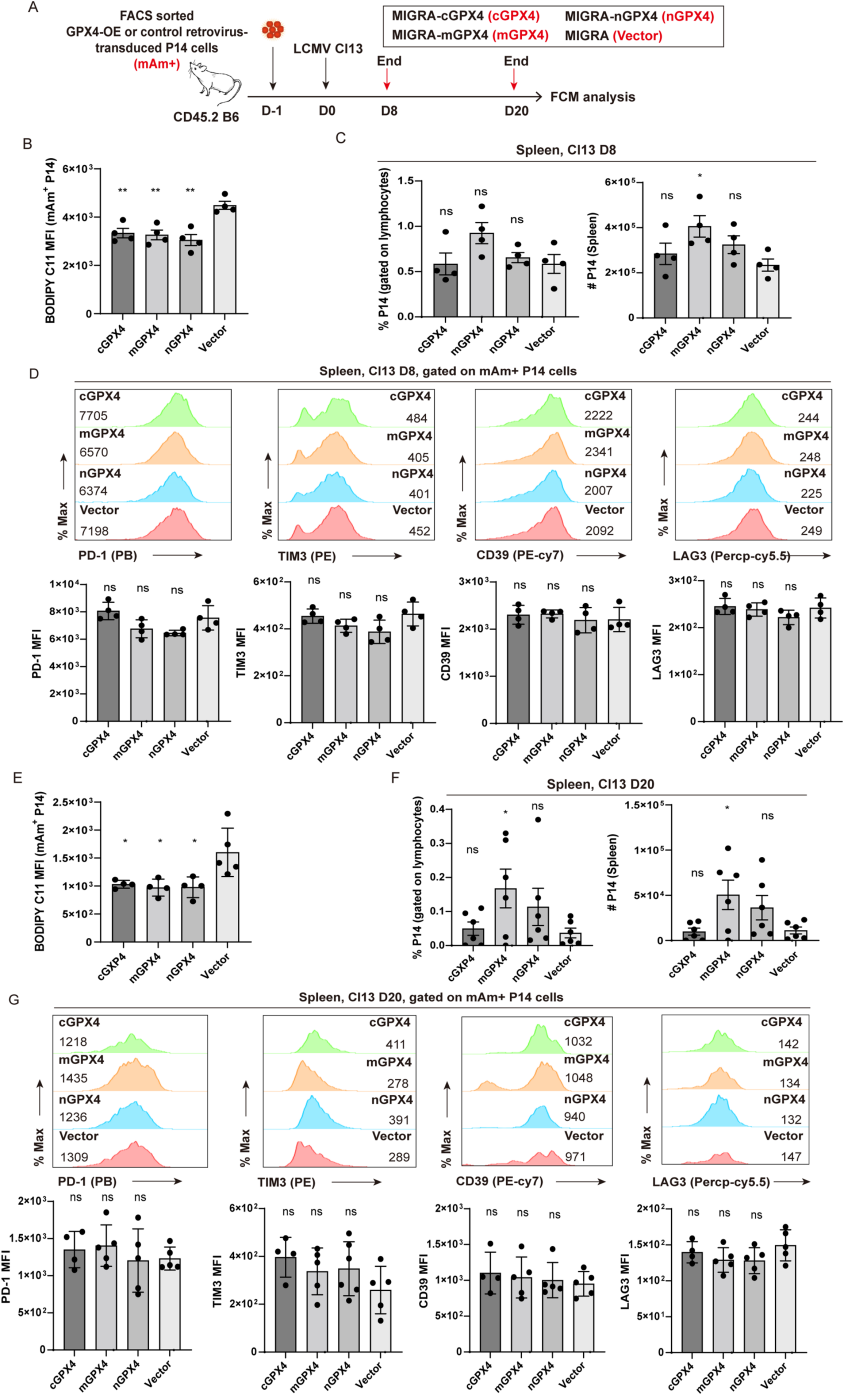
mGPX4-OE retards the decline in T_EX_ P14 cells. **(A)** Experimental setup. FACS-sorted GPX4-OE-P14 cells and vector-transduced P14 cells were adoptively co-transferred into recipient mice, followed by LCMV Cl13 infection 1 day later. Mice were euthanized at the early stage [8 dpi, **(B–D)**] and the late stage [20 dpi, **(E–G)**] of LCMV Cl13 infection, and splenocytes were subjected to flow cytometric analysis. BODIPY-C11 MFI in GPX4-OE- and vector-transduced mAm^+^ P14 cells at 8 dpi **(B)** and 20 dpi **(E)**. Frequency and cell number of mAm^+^ transduced P14 cells in GPX4-OE and vector group at 8 dpi **(C)** and 20 dpi **(F)**. Representative histogram plots and statistics of MFI of inhibitory receptors in mAm^+^ transduced P14 cells in GPX4-OE and vector group at 8 dpi **(D)** and 20 dpi **(G)**. **(B–G)** Data were collected from four to six mice per group with two independent experiments. Statistical differences were calculated between GPX4-OE group and vector group by unpaired t-test. *p < 0.05 and **p < 0.01; ns, not significant.

The above results illustrated a profound role of GPX4 and ferroptosis in the clonal deletion of virus-specific T_EX_ cells, encouraging us to examine whether GPX4 overexpression (GPX4-OE) could mitigate the decline of T_EX_ P14 cells by preventing ferroptosis. We used a set of GPX4-OE retroviruses (17) to overexpress each of three GPX4 isoforms (cGPX4, mGPX4, or nGPX4) in P14 cells, and those transduced P14 cells (mAm^+^) were transferred separately into recipient mice, followed by LCMV Cl13 infection (**Figure 7A**). Mice were euthanized at the early (D8) and late phase (D20) post-infection, respectively, and splenocytes were subjected to flow cytometric analysis (**Figure 7A**). At 8 dpi, we verified that all of the three isoforms of GPX4 can significantly reduce the intensity of BODIPY-C11 on transduced mAm^+^ P14 cells (**Figure 7B**). However, no notable discrepancies in the frequency or cell number of P14 cells were discerned between any of the three GPX4-OE groups (cGPX4, mGPX4, or nGPX4) and the control group (Vector) at this timepoint (**Figure 7C**). We also observed a comparable expression level of inhibitor receptors (e.g., PD-1, TIM3, CD39, and LAG3) on P14 cells between GPX4-OE and vector group (**Figure 7D**). Correspondingly, no notable distinctions were observed in the viral load in both lymphoid (spleen) and non-lymphoid tissues (brain) (**Supplementary Figure S6A**).

At the late stage of infection (20 dpi), our results demonstrated that GPX4-OE markedly attenuated the accumulation of lipid peroxidation (**Figure 7E**). Notably, mGPX4-OE rather than cGPX4-OE or nGPX4-OE substantially increased both the frequency and cell number of P14 cells (**Figure 7F**). Interestingly, apart from mGPX4-OE, cGPX4-OE can also significantly reduce the viral load in both lymphoid and non-lymphoid tissues, compared to control treatment (**Supplementary Figure S6B**), hinting an effective improvement of effector function induced by cGPX4-OE despite of the unaltered cell numbers. Similar to that observed at D8, no notable differences were identified in the expression of aforementioned inhibitory receptors between GPX4-OE and vector group (**Figure 7G**), suggesting that GPX4-OE mitigates the decay of T_EX_ P14 cells primarily on the replenishment of P14 cell pool.

In summary, overexpression of mGPX4 can substantially safeguard virus-specific T_EX_ cells against ferroptosis, ensuring a better persistence of T_EX_ cells, and conferring a superior control of the virus.

## 4 Discussion

During chronic infection, T_EX_ cells cannot generate functional memory population, but linger in host with damped effector function and compromised survival and proliferative ability (3, 5). Malfunctional as they are, T_EX_ cells still preserve certain cytolytic ability to limit the viral burden in hosts. In contrast to studies in the last decade preoccupying with the endeavor to revive the stemness or memory-like features of T_EX_ cells (37–41), the clonal deletion of T_EX_ cells is inadequately investigated. As is widely acknowledged, exacerbated apoptosis occurred in virus-specific T_EX_ cells, driven by the continuous TCR stimulation and inhibitory chronic inflammatory microenvironment (9–11). Whether any other forms of RCD accounting for the decline and even deletion of T_EX_ cells during chronic infection remains relatively obscure. In the present study, we demonstrated the pivotal role of ferroptosis in mediating the progressive decline of T_EX_ cells during chronic viral infection. We employed a range of molecular probes to ascertain the extent of lipid peroxidation in viral-specific T_EX_ cells. The occurrence of ferroptosis in T_EX_ cells was further corroborated by the remarkable rescue effect of the ferroptosis inhibitor Lip-1. The observation of mitochondrial dysfunction and excessive accumulation of mitochondria-derived ROS indicates a possible reason for the increased accumulation of peroxidized lipids in T_EX_ cells. In parallel, the reduced content and compromised activity of GPX4 undoubtedly unleashed ferroptosis in T_EX_ cells. We demonstrated that virus-specific T_EX_ cells with a reduction or deletion of the *Gpx4* gene endured a severer form of ferroptosis and underwent cell death at a faster rate relative to those without this genetic alteration.

Accumulating studies have shed light on the role of ferroptosis in anti-tumor function of CD8^+^ T cells in tumorigenesis (24–27) and clonal expansion and homeostasis of CD8^+^ T cells in acute viral infection (23). The present study further provides insight into the role of ferroptosis in the depletion of T_EX_ cells in chronic viral infection. Inhibiting ferroptosis via either selective inhibitor or GPX4 overexpression can markedly alleviate the cell loss of virus-specific T_EX_ cells and enhance the capability of these cells to limit viral burdens in hosts.

The current study primarily aimed to examine the involvement of ferroptosis in the depletion of virus-specific T_EX_ cells. However, due to the heterogeneity and complexity of T_EX_ cells (42–45), further investigation into the distinct subsets of T_EX_ cells, including progenitor exhausted cells, transitory exhausted cells, and terminally exhausted cells (44, 45), and the regulatory mechanisms by ferroptosis, is essential for a comprehensive understanding of T_EX_ cell dynamics during chronic viral infection. The underlying mechanisms how ferroptosis regulates the differentiation, effector function, and persistence of virus-specific T_EX_ cells also warrant further investigation and elucidation.

Given that GPX4 is a selenoprotein glutathione peroxidase fueled by the system of GSH/GSSG system (22), its antioxidant enzyme activity can be modulated in various ways. It is promising that the supplement of selenium, GSH, or even the substrates of GSH synthesis can be employed to improve the viability and persistence of T_EX_ cells. A combination therapy of ferroptosis inhibitor with an immune checkpoint inhibitor may also be promising in the treatment of chronic viral infections and even tumors.

Collectively, we affirmed that ferroptosis accounts for the clonal deletion of virus-specific T_EX_ cells, and inhibition of ferroptosis may be a feasible way to unleash a potent and synergistic therapeutic efficacy for the treatment of chronic infection and tumor.

## Data Availability

The original contributions presented in the study are included in the article/**Supplementary Material**. Further inquiries can be directed to the corresponding authors.
