# Supplementary material for: Ferroptosis exacerbates the clonal deletion of virus-specific exhausted CD8+ T cells
Source: Front Immunol. 2024 Nov 25;15:1490845. doi: 10.3389/fimmu.2024.1490845 (PMC11625764; doi:10.3389/fimmu.2024.1490845)
Supplement: Supplementary file 1 [file DataSheet1.docx]

Supplementary Material

**Ferroptosis** **Exacerbates the Clonal Deletion of Virus-specific**

**Exhausted CD8^+^ T Cells**

# Supplementary Figures


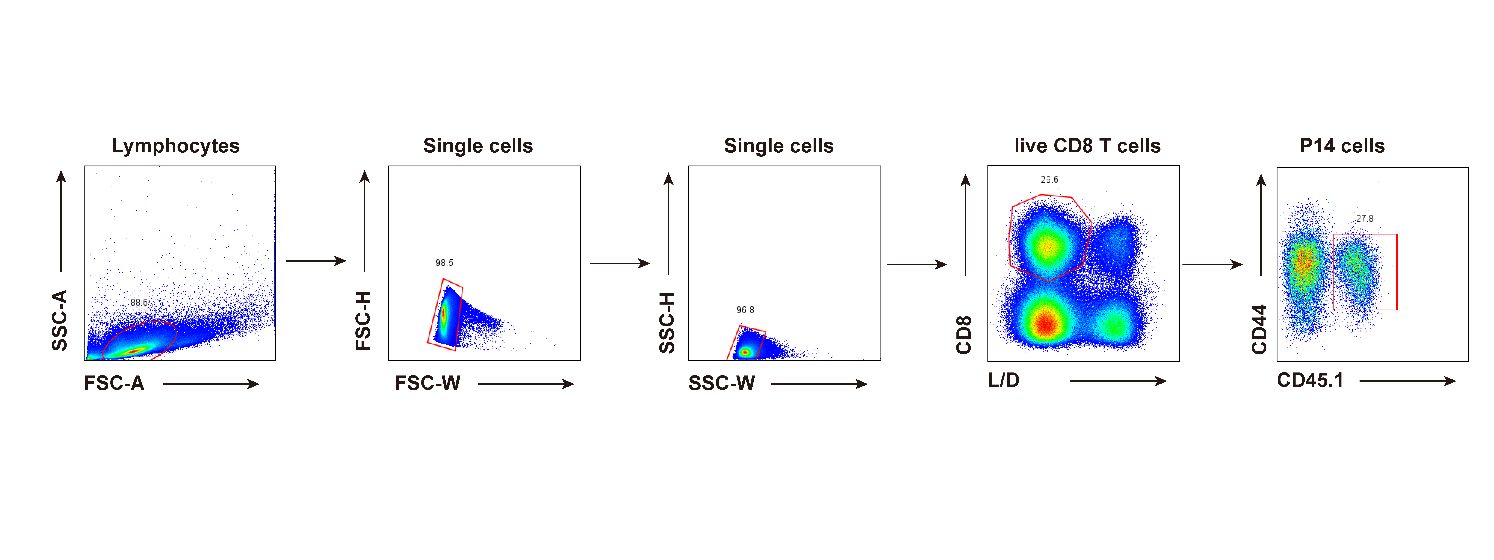
 **Supplementary Figure 1.** Gating strategy for analyzing virus-specific CD8^+^ T cells during LCMV Arm or Cl13 infection. FSC-A/SSC-A was used to gate lymphocytes. Doublets were excluded through FSC-H/FSC-W and SSC-H/SSC-W. L/D fixable dye combined with CD8 was used to gate live CD8^+^ T cells. CD44 combined with CD45.1 was used to gate P14 cells.


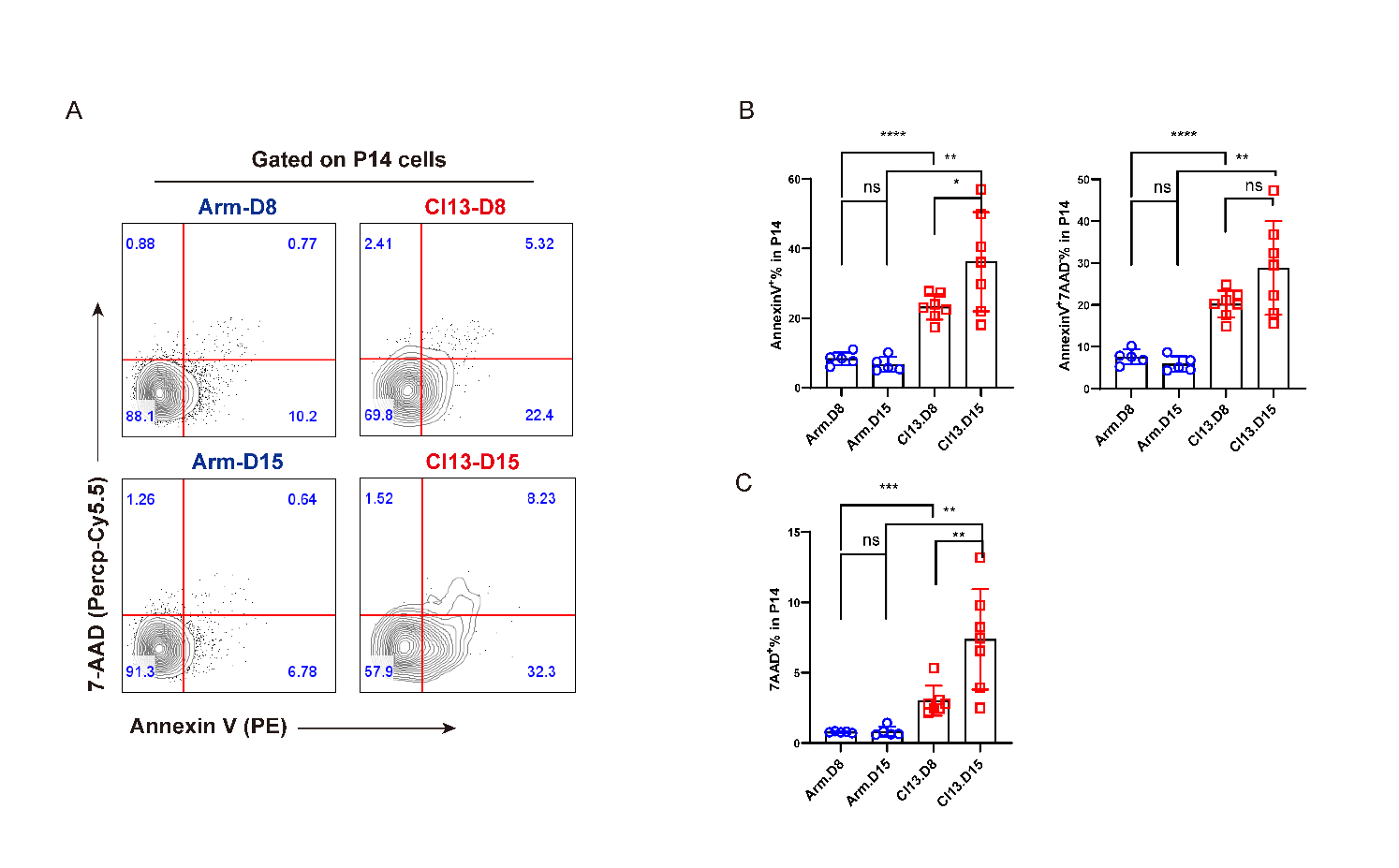


**Supplementary Figure 2.** Apoptosis partially accounts for the increased cell death of virus-specific T_EX_ cells. Experimental setup as in **Figure 1A**. (**A**) Representative contour plots showing Annexin V vs 7-AAD staining on P14 cells upon LCMV Arm or Cl13 infection, 8 and 15 dpi. Statistics revealed frequency of (**B**) apoptotic cells, including Annexin V^+^ (apoptotic) and Annexin V^+^ 7-AAD^-^ (early apoptotic), as well as (**C**) 7-AAD^+^ (dead) P14 cells. (**B**, **C**) Data were collected from 5 to 7 mice per group with two independent experiments. Statistical differences were calculated by unpaired t-test. *p < 0.05, **p < 0.01, ***p < 0.001, ****p < 0.0001, ns, not significant.


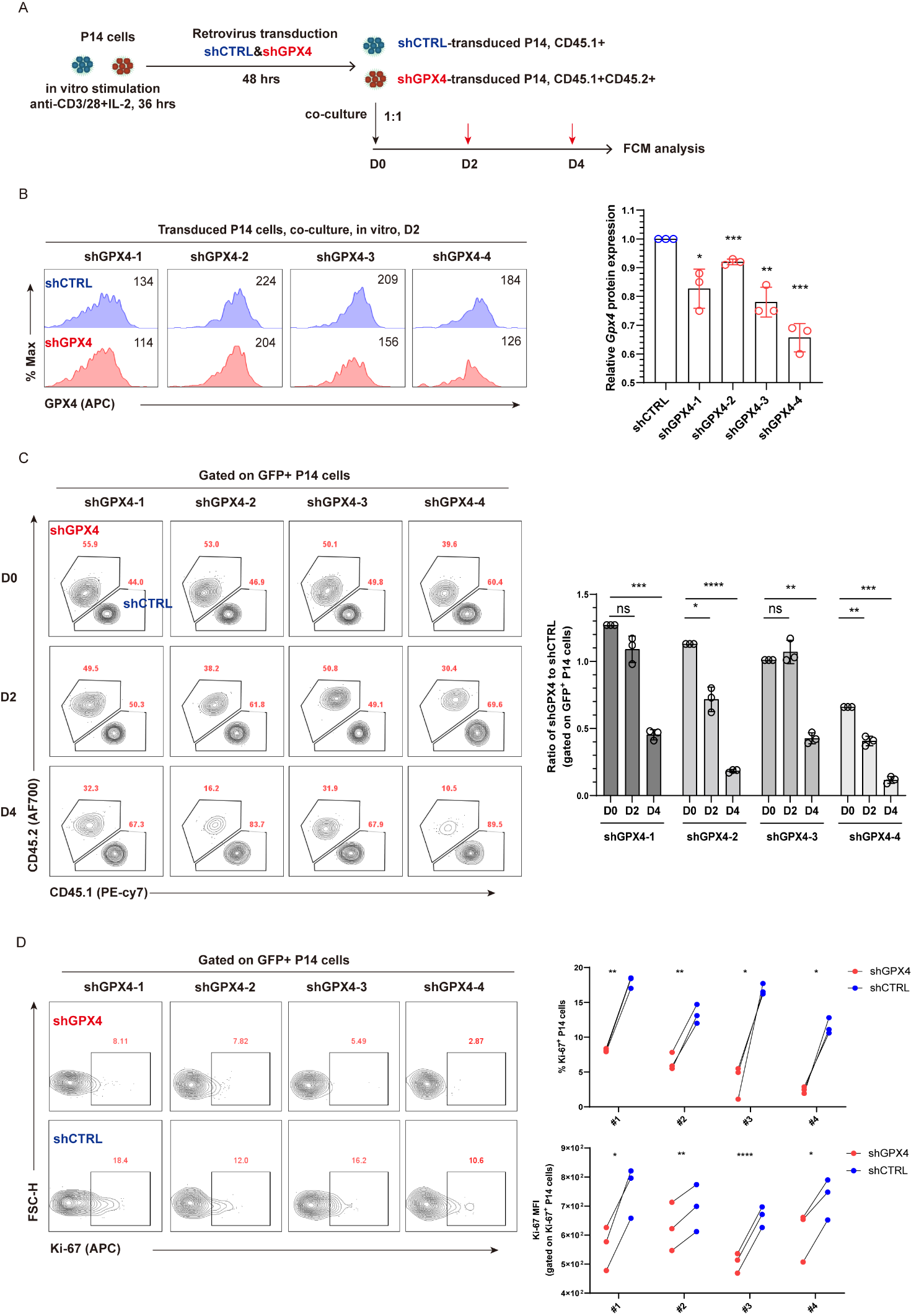


**Supplementary Figure 3.** GPX4-KD impairs P14 cell proliferation *in vitro*. **(A)** Experimental setup. P14 cells were activated in the presence with anti-CD3/28 plus IL-2 for 36hrs, then subjected to retrovirus transduction, shGPX4- and shCTRL-transduced P14 cells were mixed at a ratio of 1 to 1 after 48 hrs post transduction. Cells were analyzed at different timepoints post co-culture. (**B**) Representative histograms and statistics of protein levels of *Gpx4* via GPX4 KD at D2. (**C**) Representative contour plots and statistics of frequency of shGPX4- and shCTRL-P14 cells at different timepoints. (**D**) Representative contour plots and statistics of frequency of Ki-67^+^ P14 cells and Ki-67 MFI on Ki-67^+^ P14 cells at D4. Data were collected from 3 replicates. Statistical differences were calculated by unpaired (**B-C**) and paired t-test (**D**). *p < 0.05, **p < 0.01, ***p < 0.001, ****p < 0.0001, ns, not significant.


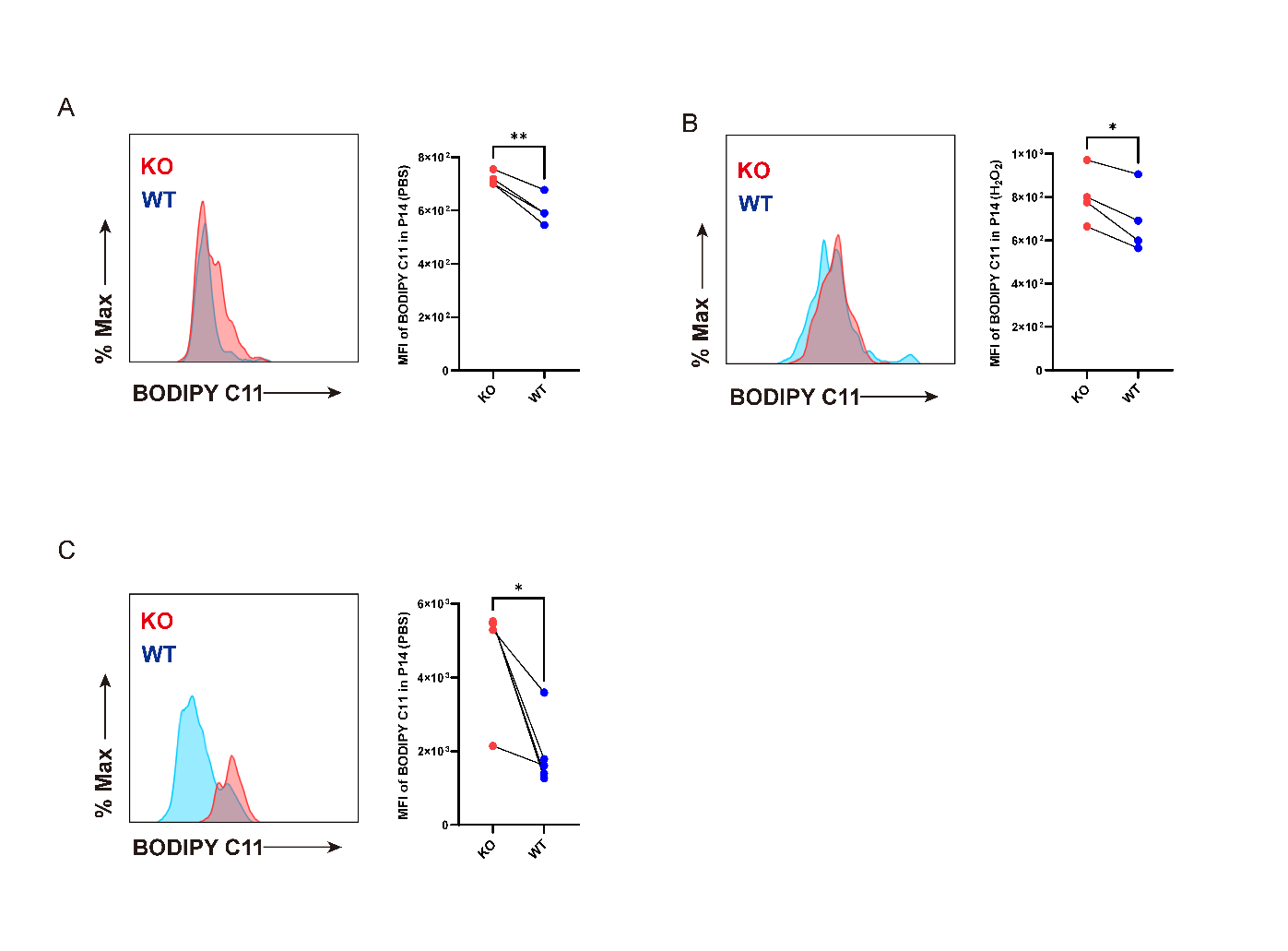
 **Supplementary Figure 4.** Lipid peroxidation in GPX4-KO-P14 and WT-P14 cells during LCMV Cl13 infection. BODIPY-C11 MFI under both physiological conditions (**A**, PBS) and excessive oxidative stress (**B,** H_2_O_2_), related to **Figure 6A-D**. BODIPY-C11 MFI under physiological conditions (**C**, PBS), related to **Figure 6E-H**. (**A-C**) Data were collected from 4 mice per group with two independent experiments. Statistical differences were calculated by paired t-test. *p < 0.05, **p < 0.01.


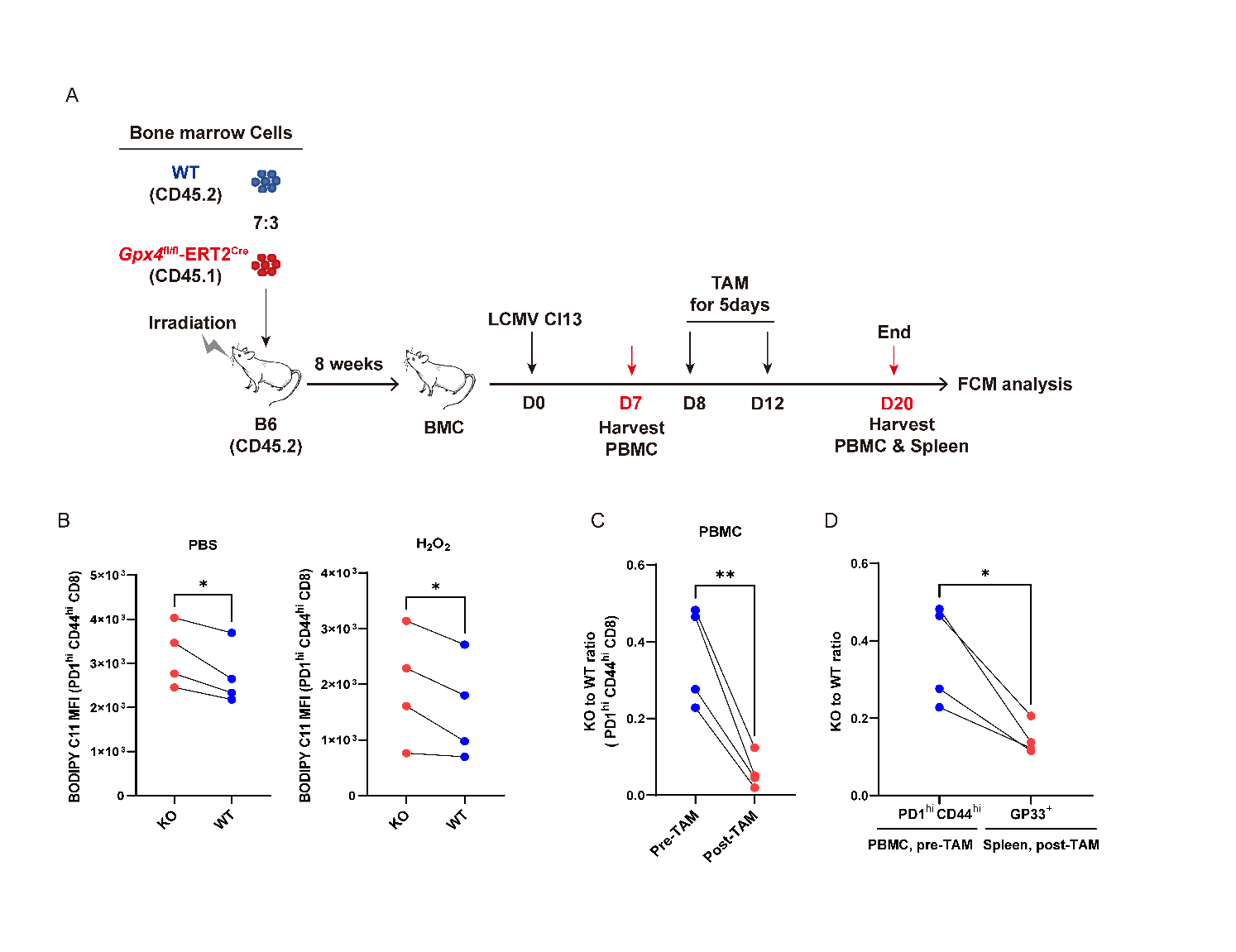
 **Supplementary Figure 5.** GPX4-KO in polyclonal and endogenous virus-specific CD8^+^ T cells in BMC models. (**A**) BMCs were generated with mixed bone marrow cells from *Gpx4*^fl/fl^-ERT2Cre (KO, CD45.1) and WT (WT, CD45.2) mice. Tamoxifen was administrated after establishment of T cell exhaustion from 8 dpi to 12 dpi, and mice were sacrificed and analyzed 8 days post treatment. (**B**) BODIPY-C11 MFI in polyclonal PD1^hi^ CD44^hi^ CD8^+^ T cells, under both physiological conditions (PBS) and excessive oxidative stress (H_2_O_2_). (**C**) Statistics of KO to WT ratio in polyclonal PD1^hi^ CD44^hi^ CD8^+^ T cells in PBMCs. (**D**) Statistics of KO to WT ratio in endogenous virus-specific GP33^+^ CD8^+^ T cells in spleens. (**B-D**) Data were collected from 4 mice per group with two independent experiments. Statistical differences were calculated by paired t-test. *p < 0.05, **p < 0.01.


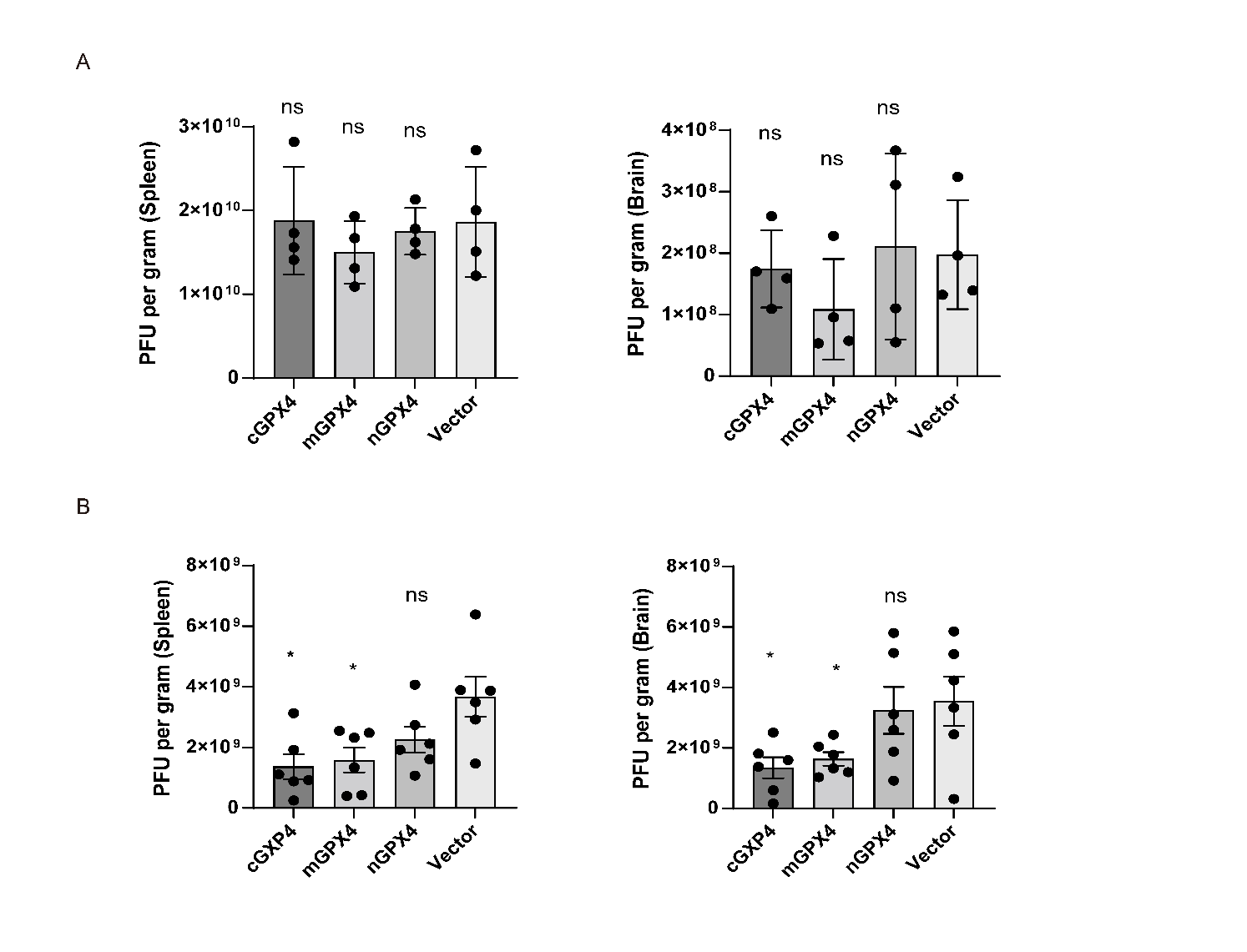
 **Supplementary Figure 6.** mGPX4-OE partially reduces viral load at the late stage of chronic viral infection. Experimental setup is illustrated in **Figure 7A.** Both spleens and brains of mice in each group were harvested at indicated weight, homogenized with Trizol, subjected to RNA isolation and qPCR determination of viral load. Viral load in lymphoid (spleen) and non-lymphoid (brain) tissues were presented as PFU per gram at 8 dpi (**A**) and 20 dpi (**B**). (**A**, **B**) Data were collected from 4 or 6 mice per group with two independent experiments. Statistical differences were calculated between GPX4-OE group and vector group by unpaired t-test. *p < 0.05, ns, not significant.
